# Hunterian Society of London

**Published:** 1889-06

**Authors:** 


### HUNTERIAN SOCIETY OF LONDON.

### SALOL AS A DRESSING.

Mr. Corner exhibited a series of cases illustrative of the antiseptic power of salol (salicylate of phenol) as a dressing for wounds, after the part had been rendered aseptic by a 1 in 20 solution of carbolic acid. Salol had a pleasant aromatic odor, could be used freely without fear of irritation or poisoning, was absorbent of moisture, and whilst drying formed a hard but friable covering. It prevented putrefaction, but did not destroy it when established. It had been used in increasing frequency for several years at the Poplar Hospital, and with excellent results in compound fractures and dislocations, also as a dressing in amputations, minor and major, and in compound comminuted fracture of the skull. The first case shown was a compound comminuted depressed fracture of the frontal bone, in which the bone was elevated, and some spicules removed, the wound being afterwards washed with 1 in 20 carbolic acid, the opening filled with salol, and a drainage-tube inserted. The dressing was undisturbed for fourteen days, remained sweet, and the wound healed on the twenty-sixth day. The temperature remained from the first under 100°. A second case treated in January, 1889, was a compound fracture of the olecranon, head of radius, and humerus, opening the elbow-joint, with considerable damage to the soft parts, the elbow having been crushed by the passage of a railway engine over it. The olecranon was split and drawn up, causing serious tension of skin, and necessitating removal of both portions. The antiseptic treatment and dressing were the same as in the previous case, but required changing after four hours and again next day in consequence of oozing through; the parts were then left undisturbed for thirty days. The temperature went up the day after the injury, and remained about 101° F. for three days, 100° F. for two days, then became normal. The other cases were shown, one a crushed compound fracture of the finger done up twenty-one days before, and not exposed since, there having been neither pain nor elevation of temperature; the other was a compound fracture of the first phalanx of the finger, dressed at the time of the accident, and undisturbed for a month, when it was found perfectly healed. It was pointed out that this was the common experience in such cases, and that even if gangrene followed, the parts remained sweet.

